# Advancements in B-Cell Non-Hodgkin's Lymphoma: From Signaling Pathways to Targeted Therapies

**DOI:** 10.1155/2024/5948170

**Published:** 2024-11-12

**Authors:** Abdullah Alfaifi, Salem Bahashwan, Mohammed Alsaadi, Ali H. Ageel, Hamzah H. Ahmed, Kaneez Fatima, Hafiz Malhan, Ishtiaq Qadri, Hussein Almehdar

**Affiliations:** ^1^Department of Biological Science, Faculty of Science, King AbdulAziz University, Jeddah 21589, Saudi Arabia; ^2^Fayfa General Hospital, Ministry of Health, Jazan 83581, Saudi Arabia; ^3^Hematology Research Unit, King Fahad Medical Research Center, King AbdulAziz University, Jeddah 21589, Saudi Arabia; ^4^Department of Hematology, Faculty of Medicine, King Abdulaziz University, Jeddah 21589, Saudi Arabia; ^5^King Abdulaziz University Hospital, King Abdulaziz University, Jeddah 21589, Saudi Arabia; ^6^Eradah Hospital, Ministry of Health, Jazan 82943, Saudi Arabia; ^7^Department of Radiologic Sciences, Faculty of Applied Medical Sciences, King AbdulAziz University, Jeddah 21589, Saudi Arabia; ^8^IQ Institute of Infection and Immunity, Lahore, Punjab, Pakistan; ^9^Prince Mohammed Bin Nasser Hospital, Ministry of Health, Jazan 82943, Saudi Arabia

**Keywords:** B-cell lymphoma, BCR, JAK/STAT, NF-kB, PI3K/AKT/mTOR, signaling pathways, targeted therapy

## Abstract

Lymphoma is the sixth most prevalent cancer globally. Non-Hodgkin's lymphomas are the majority group of lymphomas, with B cells accounting for approximately 95% of these lymphomas. A key feature of B-cell lymphoma is the functional perturbations of essential biological pathways caused by genetic aberrations. These lead to atypical gene expression, providing cells with a selective growth advantage. Molecular analysis reveals that each lymphoma subtype has unique molecular mutations, which pose challenges in disease management and treatment. Substantial efforts over the last decade have led to the integration of this information into clinical applications, resulting in crucial insights into clinical diagnosis and targeted therapies. However, with the growing need for more effective medication development, we anticipate a deeper understanding of signaling pathways and their interactions to emerge. This review aims to demonstrate how the BCR, specific signaling pathways like PI3K/AKT/mTOR, NF-kB, and JAK/STAT are diverse in common types of B-cell lymphoma. Furthermore, it offers a detailed examination of each pathway and a synopsis of the approved or in-development targeted therapies. In conclusion, finding the activated signaling pathways is crucial for developing effective treatment plans to improve the prognosis of patients with relapsed or refractory lymphoma.

**Trial Registration:** ClinicalTrials.gov identifier: NCT02180724, NCT02029443, NCT02477696, NCT03836261, NCT02343120, NCT04440059, NCT01882803, NCT01258998, NCT01742988, NCT02055820, NCT02285062, NCT01855750, NCT03422679, NCT01897571

## 1. Introduction

Lymphomas are a wide range of neoplasms that affect lymphoid tissues. The two major subgroups of lymphomas are Hodgkin lymphoma (HL) and non-Hodgkin lymphoma (NHL). NHLs are a variety of lymphoid neoplasms that form when mature B, T, and natural killer (NK) cells divide into multiple clones at different stages of development [1]. NHL constitutes around 90% of all lymphomas, with diffuse large B-cell lymphoma (DLBCL) identified as the most common subtype [2, 3]. NHLs can arise from mutations at practically every stage of lymphocyte development; therefore, numerous lymphoma subtypes exhibit similarities with their nonmalignant counterparts. These morphological and immunophenotypic similarities are crucial in the early diagnosis and overall classification of lymphomas. Different clinical presentations commonly categorize NHLs as aggressive or indolent. Aggressive B-cell lymphomas (BCLs) include DLBCL, Burkitt lymphoma (BL), and follicular lymphoma (FL), Grade III. Indolent lymphomas include small lymphocytic lymphoma/chronic lymphocytic leukemia (SLL/CLL), FL (Grades I and II), lymphoplasmacytic lymphoma/Waldenstrom macroglobulinemia (LPL/WM), marginal zone lymphoma (MZL), hairy cell leukemia (HCL), and multiple myeloma (MM) [4].

The ability to comprehend these malignants' pathophysiology has largely relied on the extensive literature on normal B-cell biology. Nevertheless, B-cell malignancies reveal gaps in our understanding of normal B-cell activity [5]. Therefore, recent years have seen a significant scientific effort to detect and comprehend molecular changes in lymphomas, leading to the documentation of a wide spectrum of genetic mutations across all types of lymphomas. It has identified numerous targetable oncogenic pathways and sparked the creation of a slew of potential new therapies [3]. In general, malignant B cells frequently modify normal B-cell signaling pathways to maintain growth and survival, either by activating or deactivating negative regulators. Therefore, imbalanced oncogenic signaling pathways and genetic mutations contribute to the development of these disorders and distinguish them from each other [5]. It is common for lymphomas to devise strategies for dysregulated signaling pathways, including the B-cell receptor (BCR) pathway. These pathways may include the phosphoinositide-3-kinase/v-akt murine thymoma viral oncogene homolog/mammalian target of rapamycin (PI3K/AKT/mTOR), the nuclear factor-kappa-B (NF-*κ*B), the Janus kinase/signal transducer and activator of transcription (JAK/STAT), the programmed death-1/programmed death-ligands (PD-1/PD-Ls), and proteins of the apoptosis cycle [6].

First-line immunochemotherapy regimens, including rituximab, cyclophosphamide, doxorubicin, vincristine, and prednisone (R-CHOP), have the potential of curing around 60% of patients. However, the diversity of genetic characteristics and phenotypes within BCL is the most significant impediment to successful targeted therapy implementation [3, 7]. Consequentially, individuals who fail first-line therapy pose a substantial therapeutic challenge, and the vast majority of these patients die as a result of their disease [7]. Interestingly, the depth of new molecular and genetic understanding has revealed numerous targetable carcinogenic pathways, kicking off the creation of a slew of potential novel therapeutic medicines [3]. Therefore, patients with lymphoid malignancies will find new hope with pathway-based targeted therapy, standard chemotherapy, a single specific targeted antibody, and immunotherapy. The logical and efficient use of pathway-related signatures in screening, prognosis, prediction, and therapeutic selection will enable the advancement of precision medicine [4, 8]. The aim of this review is to examine the BCR, PI3K/AKT/mTOR, NF-kB, and JAK/STAT signaling pathways that are out of whack and show how they contribute to the growth of BCLs. Also discussed are the targeted therapies that are either in development or have received approval. We direct readers to the comprehensive reviews referenced throughout this article for additional insights on optimal practices in lymphoma management.

## 2. Overview of Key Signaling Pathways in B-NHL

**Figure 1:**
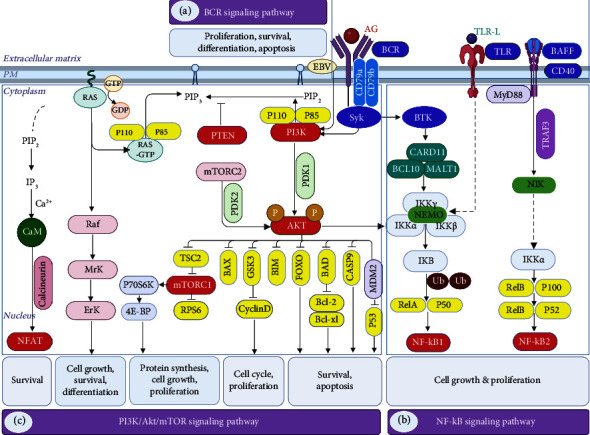
Major B-cell signaling pathways and their crosstalk. As shown in the figure, the key signaling pathways are (a) BCR, (b) NF-*κ*B, and (c) PI3K/AKT/mTOR, as well as their regulatory roles in cellular processes.4E-BP, eukaryotic translation initiation factor 4E (eIF4E)–binding protein; AKT, protein kinase B; BAD, Bcl-xl/Bcl-2-associated death promoter; BAFF, B-cell activating factor; BAX, Bcl-2-associated X-protein; BIM, Bcl-2 Interacting Mediator of cell death; CaM, calmodulin; CARD11, caspase recruitment domain family member 11; Casp9, cysteinyl aspartate specific proteinase 9; EBV, Epstein–Barr virus; ERK, extracellular signal–related kinase; FOXO, forkhead box, class O; IKK, I*κ*B kinase; MALT1, mucosa-associated lymphoid tissue lymphoma translocation protein 1; MDM2, murine double minute 2; Mrk, mitogen-related kinase; mTORC1/2, mammalian target of rapamycin complex 1/2; MYD88, myeloid differentiation primary response 88; NEMO, IKKγ, NIK, NF-*κ*B-inducing kinase; NF-*κ*B, nuclear factor-kappa-B, GSK3, glycogen synthase kinase 3; NFAT, nuclear factor of activated T cell; P53, tumor protein 53; PDK1/2, phosphoinositide-dependent protein kinase 1/2; PI3K, phosphoinositide 3-kinase; PIP2, phosphatidylinositol-(4,5)-bisphosphate; PIP3, phosphatidylinositol-(3,4,5)-trisphosphate; PTEN, phosphatase and tensin homolog, P70S6K, phosphorylation of ribosomal p70S6 kinase; Raf, rapidly accelerated fibrosarcoma; RAS, rat sarcoma; RPS6, ribosomal protein S6; RTKs, receptor tyrosine kinases; SYK, spleen tyrosine kinase; TRAF3, TNF receptor–associated factor 3; TSC2, tuberous sclerosis complex 2; TLR, Toll-like receptors; Ub, ubiquitination.

As of today, several lines of evidence suggest Bruton's tyrosine kinase (BTK); PI3K, protein kinase B (AKT), and mTOR pathways; NF-kB; and JAK-STAT pathway. Furthermore, some of the complex pathways that control growth, differentiation, and activation in normal B cells also play a pivotal role. Understanding normal signaling in B cells is crucial for identifying pathological changes in B-cell lymphoma because it provides a baseline against which abnormal signaling pathways can be assessed, which facilitates accurate diagnosis, targeted therapy, and better prognostic evaluation. Many lymphomas are characterized by specific molecular alterations, such as mutations in the BCR pathway components. Moreover, BCR inhibitors target the BCR signaling pathway, which is often dysregulated in B-cell lymphomas [9]. This section provides a detailed description of the pathways relevant to B-cell lymphomagenesis (Figure 1).

### 2.1. BCR Signaling

BCR signaling is very important for many types of NHLs, which shows how essential it is in the lymphomagenesis process. It is imperative that molecular clinicians understand the critical role of the BCR in the appropriate growth and maturation of B cells [10], so they can develop strategies to combat the lymphoma. The efficacy of BCR signaling and the activation of cofactors such as Toll-like receptors (TLR), B-cell activating factor (BAFF), and CD-40 are critical for B-cell responsiveness and survival during the germinal center response. In order for mature B cells to remain viable during periods of inactivity, they require a fundamental “tonic” BCR signal. On the other hand, antigens activate BCRs, which subsequently induce the proliferation, maturation, and production of antibodies by B cells [9, 10].

A functional BCR comprises an immunoglobulin that is membrane-bound along with two co-receptors, Ig alpha (CD79a) and Ig beta (CD79b) [3]. The BCR finds an antigen on both healthy and cancerous B cells and either sends a steady signal through the PI3K pathway or starts other pathways to work. The binding of an antigen to the BCR leads to the cross-linking of multiple receptors and morphological alterations. ITIMs of CD79a and CD79b are immunoreceptor tyrosine-based activation motifs that get tyrosine phosphorylated by Lyn and other tyrosine kinases (SFKs) from the SRC family. The phosphorylation events of spleen tyrosine kinase (SYK) initiate the materialization of a functional signalosome complex. Adaptor proteins and a variety of tyrosine kinases are involved in the complex's assembly [11]. The signal is sent by tyrosine kinases like BTK and phospholipase Cγ2 (PLCγ2). Adaptor proteins like B-cell linker (BLNK) and B-cell adaptor for phosphatidylinositol 3-kinase (BCAP) control how the signal is spread [3, 11]. Furthermore, phosphatases play a role in BCR signaling regulation. SH2-containing protein tyrosine phosphatase 1 may inhibit SRC and SYK. In contrast, the SH2-containing protein tyrosine phosphatase 2 (SHP2) is important in signaling, particularly in the ERK pathway [9, 11]. In general, the complex network of proteins that are involved in BCR signaling guarantees a well-regulated and balanced immune response. Various autoimmune diseases and malignancies can result from the dysregulation of these pathways. Understanding the precise mechanisms of BCR signaling can provide valuable insights for therapeutic interventions that target these conditions.

### 2.2. PI3K–AKT–mTOR Pathway

Numerous biological functions, such as metabolism, angiogenesis, cell proliferation, survival, and differentiation, depend on the PI3K/AKT/mTOR pathway [12]. After the BCR is depleted, activation of the PI3K pathway is enough to sustain the survival of mature B cells at rest. Consequently, it is proposed that the primary component of the tonic BCR signaling is the PI3K signal transduction. Moreover, even though PI3K itself is commonly altered, the tonic signal is crucial for a large number of B-cell malignancies. The adaptor protein BCAP transfers the BCR signal to a PI3K heterodimer made up of a catalytic and a regulatory [13]. Additionally, multiple effectors are implicated. PI3K and BTK collaboratively activate NF-*κ*B. In contrast, AKT serves as the link between PI3K and mTOR pathways. It turns on phosphoinositide-dependent kinase-1 (PDK1), which then binds and phosphorylates AKT by working with mTOR complex 2 (mTORC2). After that, AKT phosphorylates mTORC1 and turns off Forkhead/winged helix box class O (FOXO), which helps cells move through the cell cycle and stay [14].

Two well-known products such as ribosomal S6 kinase (S6K) and the eIF4E binding protein (4E-BP1) also modulate the mTORC1. A cascade of events follows leading to protein synthesis, nutrient response, and several additional functions essential for rapid growth. Additionally, the negative feedback mechanisms that diminish upstream PI3K-AKT signaling include the inactivation of insulin receptor substrate 1 (IRS-1), the suppression of mTORC2. The mTORC pathway is also dependent on S6K, and the phosphorylation of the adaptor protein glycine-rich RNA-binding protein 10 (GRB10) [12, 13, 15]. Understanding the mechanisms that govern mTORC1 signaling is critical for developing targeted therapies. Investigating the consequences of reduced PI3K-AKT signaling may provide insights into potential treatment strategies for BCLs.

### 2.3. NF-*κ*B Pathway

The NF-*κ*B pathway plays a crucial role in B-cell proliferation and survival by counteracting apoptotic signals [16]. Different types of BCLs have abnormal NF-*κ*B activation, which leads to increased cell cycling and decreased apoptosis. In addition to this, PLC2, BTK, and the adapter protein BLNK promote the activation of the canonical NF-B pathway. Along with diacylglycerol (DAG) and more calcium moving inside cells, PLCγ2 activity causes the classical isoform PKCβ to phosphorylate CARD11 [17]. BCL-10 initiates the activation of mucosa-associated lymphoid tissue lymphoma translocation protein 1 (MALT1). Following multimerization with CARD11, the complex recruits TNF receptor–associated factor 6 (TRAF6) and TAK1. A process involving ubiquitin is used by TRAF6 to connect with IKK, while TAK1 helps IKK's activation cycle phosphorylation. The inhibitory protein I*κ*B is phosphorylated by IKK. This causes it to be polyubiquitinated and then broken down by proteases, and then, transcription factor NF-*κ*B moves into the cell [18, 19].

The different NF-*κ*B pathway turns on IKKs, which directly phosphorylate and partially break down certain NF-*κ*B subunits. NF-*κ*B1 (p50), NF-*κ*B2 (p52), c-Rel, RelA (p65), and RelB (p52) form heterodimers. Nucleus-localized heterodimeric NF-B transcription factors initiate target gene transcription. NF-*κ*B target genes encompass cell cycle regulators, antiapoptotic, inflammatory, and immunoregulatory molecules, along with negative feedback regulators that impede upstream pathway activation. The NF-*κ*B pathway turns on proteins in the BCL-2 family that stop cell apoptosis, as well as MYC and cyclin D1, which control the cell cycle. Immunoregulatory cytokines are made up of interleukins (ILs) like IL2, IL6, and IL10. These ILs activate growth receptors either directly or indirectly [5, 20]. NF-*κ*B functions as a critical regulator of immune responses and body homeostasis maintenance. Its complex role in multiple pathways underscores its significance in both normal physiological functions and pathological conditions such as cancer. In lymphomas, dysregulation of NF-*κ*B signaling can result in uncontrolled cell proliferation and inflammation. Understanding the intricate role of NF-*κ*B in immune responses is critical for the development of targeted therapies for various lymphomas.

### 2.4. JAK-STAT Signaling

The JAK and STAT pathways are primarily activated by ILs. Both autocrine and paracrine mechanisms release them. The regulation of cell proliferation, survival, differentiation, and immune response is contingent upon the JAK-STAT pathway. Elevated serum levels of various ILs accompany BCR signaling in NHLs. When there are changes in the NF-*κ*B pathway in ABC-DLBCLs, IL-6 or IL-10 is made, which raises the activity of growth factors [21, 22]. Cytokine binding may cause basally inactivated cognate receptors to dimerize. The activation of intracellular JAKs leads to the cross-phosphorylation of the receptor. JAKs have the potential to phosphorylate STAT proteins as a result of their binding to them. For example, the binding of IL6 or IL10 to the receptor in NHL transmits a signal to STAT3 via JAKs [21]. JAKs may also activate the PI3K, mTOR, and MAPK pathways; however, their activity is halted by either phosphatase activation or receptor internalization [22, 23].

Furthermore, phosphorylated STATs dimerize and shift to the nucleus, functioning as transcription factors. In addition, STATs control suppressors of cytokine signaling (SOCS) transcription, as well as proliferative and surviving genes. SOCS 1–7 are part of the negative feedback loops in this pathway. They halt STAT signaling by directly linking to JAK, thereby preventing more STAT phosphorylation. Furthermore, STAT protein inhibitors (PIAS) interact directly with STATs to impede their transcriptional function [22]. Researchers have identified STAT signaling pathway dysregulation as a factor in various cancers, including lymphoma. Understanding how SOCS and PIAS regulate this pathway is critical for developing targeted B-cell NHL therapies.

### 2.5. Apoptosis Signaling Pathway

Apoptosis refers to the PD of a cell, distinguishing it from other forms of cell death, including autophagy, necroptosis, netosis, pyroptosis, necrosis, and ferroptosis. Apoptosis induces various morphological changes in the cell, including a reduction in the size of the mitochondria and nuclei. Apoptosis is characterized by a reduction in cell function and size, a phenomenon referred to as “blebbing.” During blebbing, certain regions of the cell membrane encapsulate the cellular contents. Phagocytosis rapidly removes apoptotic cells from the site, thereby mitigating the robust inflammatory response. A TNF-regulated extrinsic death receptor pathway can initiate apoptosis. The intrinsic mitochondrial pathway can also mediate this process. The mitochondria that are helped by tBID (truncated BH3-interacting domain protein) are where these pathways come together [24]. For example, tumor necrosis factor receptor 1 (TNFR1) and Fas are two death receptors that are activated when ligands bind to them. In the beginning, Fas connects to FADD (Fas-associated via death domain) and then joins with caspase 8. This makes a signaling complex that can start apoptosis. This facilitates apoptosis by directly activating caspase 3 and caspase 7, or indirectly triggering caspase pathways reliant on mitochondrial mechanisms [25]. Compared to the Fas-mediated pathway, TNFR1-mediated apoptosis is more conducive to the incorporation of the intricate TRADD (TNFRSF1A-associated via death domain) and TRAF2. This causes cell death by activating NF-*κ*B via IKK [26]. Hypoxia, DNA damage, and oncogenic stress trigger apoptosis more swiftly in the intrinsic apoptotic pathways. In both upstream and downstream cascades, p53, DNA checkpoint proteins, and BCL2 family members work together to control and send signals along this pathway. This ultimately facilitates the activation of caspase 3 and caspase 7, resulting in results similar to those obtained via the extrinsic route [25]. Besides apoptosis, MYC transcription factors directly and indirectly govern cell proliferation, metabolism, differentiation, and oncogenes. Consequently, the majority of cancers arise from C-MYC dysregulation. Rapidly proliferating cancer cells inherently produce increased levels of oncogenic MYC, resulting in diminished genomic stability. This indicates that late-stage or recurring cancer cells, which are likely to have elevated MYC levels, must possess increased DNA damage. In advanced cancer cells, chemotherapy and radiation induce genomic instability, prompting oncogenic MYC to develop an efficient DNA repair mechanism. Because of its involvement in carcinogenesis, MYC inactivation should reduce cancer cell survival [27].

In summary, B-cell neoplasms frequently disrupt the normal signaling pathways used by B cells for antigen recognition. This ensures the continuous activation of pathways that facilitate survival. BCR is crucial for the regulation of a variety of processes in normal B cells, such as proliferation, differentiation, apoptosis, and cell migration. Proximal BCR signaling activates the PI3K, NF-B, NFAT, and MAPK pathways. Other signaling events occur at greater distances. Signal transmission through the MAPK pathways involves the activation of multiple pathways, including MAPK, ERK, JNK, and p38 [23]. All BCR-induced pathways facilitate the survival and proliferation of B-lymphocytes.

## 3. Mechanisms and Implications

HL and NHLs distinguish BCLs, a heterogeneous category of cancers, based on their unique clinical, phenotypic, and genetic characteristics. Mature B cells produce BCLs as a result of the germinal center reaction. The variety of lymphomas represents the range of stages in the germinal center response. The classification of lymphoma may be based on the genetic alterations causing aberrant signal transduction and the B cell's origin state [28]. In order to guarantee their unchecked growth and survival, BCLs often take advantage of the mechanisms that control normal B-cell differentiation and activation. B cells are particularly prone to developing cancer because of the mechanism of antibody diversification, which may result in chromosomal translocations and other alterations that might lead to cancer. B-cell malignancies often interfere with the signaling pathways used by healthy B cells to identify antigens, leading to the chronic activation of prosurvival pathways [5]. Mutations that cause abnormal signaling by altering cascades and feedback mechanisms have a significant impact on cancer progression and survival [17]. Since lymphomas and their mutations are heterogeneous, understanding the molecular pathways that cause and progress carcinogenesis is crucial to improving therapeutic options and reducing resistance and recurrence [28]. Many types of B-cell cancer are very sensitive to kinase inhibitors that block BCR signaling, even though different subtypes have different mutations and different clinical outcomes. BCR signaling targeted therapy is a novel treatment option for various B-cell malignancies.  Table 1 lists the genetic mutations that cause signaling problems in common types of B-NHLs, including DLBCL, CLL/SLL, FL, MZL, mantle cell lymphoma (MCL), and BL.

### 3.1. DLBCL

DLBCL is a neoplasm that features medium- to large B-lymphoma cells that display a diffuse proliferation pattern. DLBCL encompasses distinct condition entities defined by specific clinical, abnormal, and biological characteristics. We diagnose DLBCLs not otherwise specified, commonly referred to as DLBCL, when they fail to fit into a specific classification. DLBCL represents the most prevalent subtype of B-lymphoma, comprising 25%–35% of all B-NHLs [2, 3]. The R-CHOP regimen, comprising rituximab, cyclophosphamide, doxorubicin, vincristine, and prednisone, serves as the standard treatment for patients with DLBCL, achieving a cure rate of 50%–70% among those who receive it. Nonetheless, the remaining patients either exhibit resistance to R-CHOP treatment or experience disease relapse following a complete response (CR). Comprehending the biology of DLBCL is crucial for identifying individuals who are unlikely to attain remission with R-CHOP and for exploring other treatment strategies. The discovery of different biological subtypes of DLBCL is a big step forward in research. The main way to classify DLBCL is into subtypes that are like germinal center B cells (GCB) or activated B cells (ABC). The two subtypes exhibit unique genomic profiles and correlate with varying clinical outcomes [29].

DLBCL genetics are complex; therefore, we focus on significant genetic mutations and their potential contributions to DLBCL pathogenesis. These target gene expressions significantly enrich ABC-DLBCL, a subtype of DLBCL, suggesting a critical role for deregulated constitutive NF-B. Genetic mutations in DLBCL drive a common pathway of deregulation, which in turn leads to constitutive NF-B activation. It has been demonstrated that mutations target the NF-*κ*B signaling complex of CARD11, oligomerized BCL10 and MALT1, regulator A20, and the signaling complex of the BCR. Missense mutations in CARD11, particularly in the coiled-coil domain, are essential for the activation of NF-B, which in turn facilitates oligomerization [5, 29]. Twenty percent of ABC-DLBCLs deleted or mutated one negative regulator, A20. More than 20% of ABC-DLBCLs have somatic mutations in the BCR signaling proteins CD79A and CD79B. These mutations cause BCR signaling to stay active for longer, which starts complicated processes that are needed for tumor cells to grow and stay alive. ABC-DLBCL causes trisomy 3, 6q deletion, 18q amplification, 9 interstitial deletion, and 19 amplification. Certain gene changes imply secondary, substantially unregulated pathways. Most ABC-DLBCL patients lose just p16INK4A, indicating a dismal fate [30]. By turning PIP3 into inactive PIP2, the tumor suppressor PTEN may reverse this process. PTEN loss increases PIP3, which releases PI3K/AKT pathway inhibition. This route promotes cell growth, angiogenesis, and proliferation [13, 15].

Implications: Numerous studies have demonstrated that deletion or expression of PTEN has varying prognostic implications in DLBCL [6]. The study by Pfeifer et al. found that over 55% of GCB-DLBCL patients had deregulation of the pathway involving PI3K/AKT as a result of PTEN loss, compared to just 14% of non-GCB-DLBCL cases. In addition, 248 individuals with primary DLBCL who had this dysregulation had a dismal prognosis [48]. Contrarily, two large-scale studies of DLBCL patients found that PTEN mutation and deletion did not significantly affect clinical outcomes. Findings from this study provide credence to the idea that the MYC and PI3K/AKT/PTEN signaling pathways are independent and not sequential [8, 49]. In this subtype of DLBCLs, therapeutic interference with apoptosis protection is of the utmost importance. Interactions between MYC and certain miRNAs can change the expression of target genes. This throws off the balance of MYC signaling networks, especially between functions that cause cell death and functions that cause cell growth. This may indirectly lead to GCB-DLBCL [27, 31, 32]. MYC transcription factors regulate cell proliferation, metabolism, differentiation, and cancer genes directly and indirectly. Therefore, most malignancies including BCLs result from c-MYC deregulation. Rapidly growing cancer cells naturally make more oncogenic MYC, which makes the genome less stable. Cancer cells with high MYC levels tend to be in the late stages or return after treatment, which suggests that there must be more DNA damage in these cells. In advanced cancer cells, radiation and chemotherapy cause genomic instability, which oncogenic MYC compensates for by developing a highly efficient DNA repair system. Inactivating MYC should reduce cancer cell survival due to its role in carcinogenesis [27].

Mechanisms: It is essential to examine the mechanisms that trigger genetic mutations in DLBCL in order to comprehend the disease's pathogenesis. About 80% of the mutations in DLBCL are due to age-related spontaneous deamination, according to an examination of mutational signatures. DLBCL causes changes in DNA via processes that are exclusive to B cells. The IGH locus may experience breakpoints caused by RAG1 and/or RAG2, which can result in gene fusions like IGH-BCL2. Only single-stranded DNA contains the enzyme activation-induced cytidine deaminase (AID). It changes cytosine residues to uracil residues, which lets somatic hypermutation and class-switch recombination happen. In addition to immunoglobulin DNA variables, AID targets other elements and switches recombination patterns. It targets actively transcribed genes, such as BCL6 and MYC, and interferes with their function. The shift region of IGH is where most breaks in the translocations of IGH-BCL6 and IGH-MYC occur. This suggests that AID-facilitated class-switch recombination is the process that leads to these translocations. AID induces BCL2 breaks in pre-B cells, although AID expression is lower than that observed in GCB. The RAG complex is not implicated due to the presence of a GC-rich motif within the BCL2 breakpoint region, which is not a target for the RAG complex. In DLBCL, AID-mediated off-target hypermutation may play a role in the development of oncogenic mutations. More than 50 percent of patients diagnosed with DLBCL exhibit hypermutations in nonimmunoglobulin genes. AID predominantly induces mutations in BCL2, PIM1, SGK1, and IGLL5, as demonstrated by a 2018 study. AID-induced mutations primarily manifest as single nucleotide substitutions, whereas deletions and duplications occur with lesser frequency. The data indicate a propensity for transversions as opposed to transitions, with the RGYW pattern receiving particular attention, implying its role in somatic hypermutation [29]. In DLBCL, MYC acts as a genetic and protein expression-based independent predictive marker. Recently, “high-grade BCL with MYC and BCL2 and/or BCL6 rearrangements” (HGBL-DH) was officially classified as a distinct entity by the World Health Organization (WHO). Numerous cancer centers recommend dose-intensive chemotherapy regimens due to the extremely poor prognosis associated with these lymphomas [50]. It is important to do further research on MYC mutations in BCL, particularly in refractory and relapsing pathways.

Clinical outcomes among individuals with DLBCL-administered R-CHOP exhibit significant heterogeneity, underscoring the need for further research to create more effective prognostic tools. Determining various genetic abnormalities will enhance risk stratification for individuals with DLBCL. Numerous studies have utilized genetic abnormality data to create prognostic models for DLBCL, potentially outperforming risk stratification methods that only use standard clinical indicators [29].

### 3.2. CLL/SLL

CLL is a prevalent B-cell malignancy characterized by significant heterogeneity in its clinical course, which can vary from indolent forms requiring no immediate treatment to rapidly progressive variants that exhibit therapeutic resistance [51].

CLL Genetics: Whole-genome sequencing (WGS) has enabled the characterization of the genomic terrain encompassing CLL. In total, 11 recurrent somatic copy number variations and 44 recurrently mutated genes have been identified. Some of these genes are PTPN11, ZNF292, IKZF3, SF3B1, FBXW7, ATM, TP53, FBXW7, SF3B1, CHD2, POT1, ARID1A, BXW7, and PTPN3.

Implications: The central pathways involved in CLL are RNA processing and export, MYC expression, and MAPK signaling. Furthermore, these analyses frequently implicate proteins that play a crucial role in the destruction of DNA signaling and repair. Patients who are secondary resistant to chemotherapy that damages DNA have higher levels of del(11q) and del(17p), as well as somatic mutations in ATM and TP53 that stop them from working. Underlying genes 9p13 contain an enhancer that, when mutated, can inhibit the activity of the B cell–specific transcription factor, PAX5 [33, 36]. BCR signaling is critical in the development of CLL. The BCR engages and signals, triggering pathways that control the destiny of normal or leukemia B cells. When the BCR is activated, it brings in kinases like SYK and SRC kinase LYN. These kinases change the shape of immunoreceptor tyrosine-based activation motifs (ITAMs) on CD79b and CD79a, two helper proteins in the BCR complex [52]. It is possible for adaptor proteins and other kinases, such as BTK and PI3K, to join the BCR accessory molecule complex when CD79a and CD79b ITAMs are phosphorylated.

Clinical Outcomes: Activation of these signaling molecules activates AKT/mTOR, NF-*κ*B, and/or ERK. ZAP-70 might improve BCR signaling in CLL not by making more kinases active, but by making it possible for more kinases, like SYK, to join the BCR complex [5, 24]. In any event, ZAP-70's relationship with aggressive illness is most likely due to the increased signaling capacity it provides [37].

### 3.3. BL

BL was first identified by Denis Burkitt in 1958 as a very aggressive BCL in children from Africa. BLs constitute 40 to 50 percent of lymphomas in pediatric populations, while they represent only 1 to 2 percent of lymphoma cases in adults [53]. BLs have uniform, extensively proliferating cells, as well as a starry-sky aspect. This is because tissue macrophages eat apoptotic debris. EBV infection is associated with all endemic cases, 15% of cases that occur sporadically, and 40%–50% of immunodeficiency-associated BLs, although the implications are still under investigation [55].

BL Genetics: BL frequently alters *the gene* MYC, with changes occurring in 70% of cases. These mutations commonly impact the transactivation domain of the gene. A significant number of these mutations are probably caused by AID, due to the close proximity of the immunoglobulin and MYC locus in BL. Certain mutations of the transactivation domain increase the capacity to convert cells and specifically hinder their ability to activate the proapoptotic protein Bim from the Bcl2 family. These mutants show a separation of MYC's pro-proliferative function from its ability to trigger cell death. BL frequently exhibits TP53 inactivating mutations, with a prevalence of 35%. The selection of these mutations is likely due to the fact that when MYC is overexpressed in primary cells, it triggers apoptotic pathways that rely on TP53 [55]. There are reports of more intricate karyotypes and numerous genetic abnormalities such as *p16, p53*, *RBL2/p130, BAX*, and *BCL6*. However, the fact that some features of this disease are similar to DLBCL may lead to unique molecular changes that affect the control of important cellular pathways, making this disease molecularly distinct. The preponderance of these cases comprises the novel class of “BCL unclassifiable with characteristics intermediate between DLBCL and BL,” which is also referred to as “double-hit” lymphomas. These instances are distinguished by the rearrangement of both MYC and BCL2, and, to a lesser extent, MYC and BCL6 as well.

Clinical Outcomes: The patients have a poor clinical prognosis, and the overexpression of BCL2 may potentially significantly protect against apoptosis. BL has been associated with microRNAs, and the relationship between miRNAs and MYC is intricate [32, 55].

### 3.4. FL

FL is characterized by the translocation t(14; 18) (q32; q21)/IGH::BCL2, which is prevalent in 80%–85% of affected individuals. In contrast, t(2; 18) (p12; q21)/IGK::BCL2 and t(18; 22) (q21; q11)/BCL2::IGL are less frequent variant translocations.

FL Genetics: The overexpression of the BCL2 protein occurs due to the regulation of the BCL2 oncogene by immunoglobulin genes, which is the result of these translocations. In FL, deletions of chromosome regions 1p (15%–20%), 6q (20%–30%), 10q/PTEN (20%), and 13q (15%) are common secondary changes that make FL unique. Additionally, there are gains observed in 1q (25%), 2p (25%), 8q (10%), 12q (20%), and 18q (30%). Additionally, we note trisomies of chromosomes 7 (20%), 18 (20%–30%), and X (20%). Reports also reveal recurrent copy-neutral losses of heterozygosity (CN-LOH) at 1p (30%), 6p (20%), and 16p (20%–25%). The genes most often changed by somatic variants are those that control epigenetics and transcription (KMT2D, CREBBP, EP300, EZH2), JAK-STAT and NOTCH signaling (SOCS1, NOTCH1, NOTCH2), immune evasion (TNFRSF14), the BCR/NF-*κ*B pathway (CARD11, TNFAIP3, CD79A, CD79B, MYD88), and cell growth and death (BCL2, TP53). Ten to twenty percent of patients develop an aggressive lymphoma, most commonly DLBCL, after histological transformation of FL. During the process, genomic changes happen, such as the addition of oncogenes like REL/BCL11A (2p16), BCL6 (3q27), and MYC (8q24), and the deactivation of tumor-suppressing genes such as TP53 (17p13) and CDKN2A/B (9p21). While the t(14; 18) (q32; q21) is widely regarded as the principal genetic occurrence in FL, the BCL2 translocation (BCL2-FL) is absent in approximately 10%–15% of all FL patients [33].

### 3.5. MCL

MCL is an uncommon kind of cancer that originates from B cells and has diverse clinical characteristics.

MCL Genetics: Molecularly, the most prevalent type is the conventional MCL (cMCL), which is defined by the lack of IGHV mutations, high SOX11 expression, and a lot of genetic complexity. The leukemic non-nodal MCL (nnMCL) is a less frequent subtype that originates from a cell with germinal center reaction. It is characterized by mutant IGHV genes, a lack of SOX11 expression, and minimal genetic changes. Every rearrangement results in a constant and excessive production of cyclin D1 protein. Approximately 5% of MCL cases do not exhibit CCND1 overexpression and rearrangement. They exhibit alternative primary rearrangements involving the CCND2 or CCND3. These rearrangements occur more often with IG light chains than with IGH. Somatic mutations in MCL differ according to the specific molecular subtype. cMCL has a lot of changes in genes that control the cell cycle, the response to DNA damage, epigenetic modification, the NOTCH pathway, and NF-kB signaling pathways. Mutations in the TP53 gene and an elevated level of genomic complexity correlate with worse prognosis in both subtypes of MCL [33].

Mechanisms: Currently, various studies suggest the repetitive but less frequent gain of functional mutations in Notch1 and Notch2 as found in B-cell carcinomas, such as DLBCL, MCL, SMZL, CLL, and infrequently in FL. There is also the possibility of the existence of nonmutational Notch initiation mechanisms [56]. Some data also suggest the participation of Hedgehog signaling in the survival or proliferation of cancerous stem cells in certain hematopoietic malignancies, including acute leukemias, chronic myelogenous leukemias, and plasma cell myeloma. The secretion of Hedgehog ligands occurs inside the stromal cells in a paracrine model that contributes to the initiation of Hedgehog signals in malignancies of low-grade B cells, such as MCL, CLL/SLL, and plasma cell myeloma [57].

### 3.6. MZLs

MZLs represent the second most common category of low-grade BCLs. The location of the affected area divides this entity into three subgroups: extranodal (EMZL) of MALT, splenic (SMZL), and nodal (NMZL).

MZL Genetics: These entities differ in their biological and genomic features; however, all subtypes share certain cytogenetic alterations, such as gains of chromosomes 3/3q and 18/18q. These changes occur in approximately 20%–30% and 20%–25% of instances, respectively. Numerous genetic factors frequently activate the NF-kB signaling pathway in MZL [33].

Mechanisms: 10 to 30 percent of the time, deletions, and/or somatic mutations are what turn off TNFAIP3, which is a negative regulator of NF-kB. Somatic mutations in SMZL and NMZL (3%–5%), primarily translocations in EMZL, can impact BIRC3, while somatic mutations in CARD11 (3%–7%) frequently affect it. All MZLs have somatic mutations in genes that control epigenetics. These genes are KMT2D, CREBBP, TBL1XR1, ARID1A, EP300, EZHZ, and TET2. Some entities have a larger concentration of specific mutated genes, such as KMT2D in NMZL, whereas EZH2 is essentially absent in SMZL [33].

In conclusion, various subtypes of B-NHL exhibit unique molecular and genetic alterations that markedly affect their pathogenesis, clinical behavior, and therapeutic responses. Customizing treatment according to these molecular characteristics enhances disease management and improves patient outcomes. The molecular subtype of DLBCL influences treatment response and prognosis. Standard therapies demonstrate reduced efficacy in ABC-type DLBCL compared to GCB-type DLBCL. The aggressive characteristics of MCL and CLL, which can evolve into a more severe form, frequently necessitate intensive treatment protocols, including high-dose chemotherapy and targeted therapies such as BTK inhibitors (e.g., ibrutinib). The treatment response may present challenges, and relapse of the disease is frequently observed. The following section will provide an overview of targeted therapies that are presently utilized in clinical settings, as well as those under development for BCLs.

## 4. Current and Emerging Therapies

**Figure 2:**
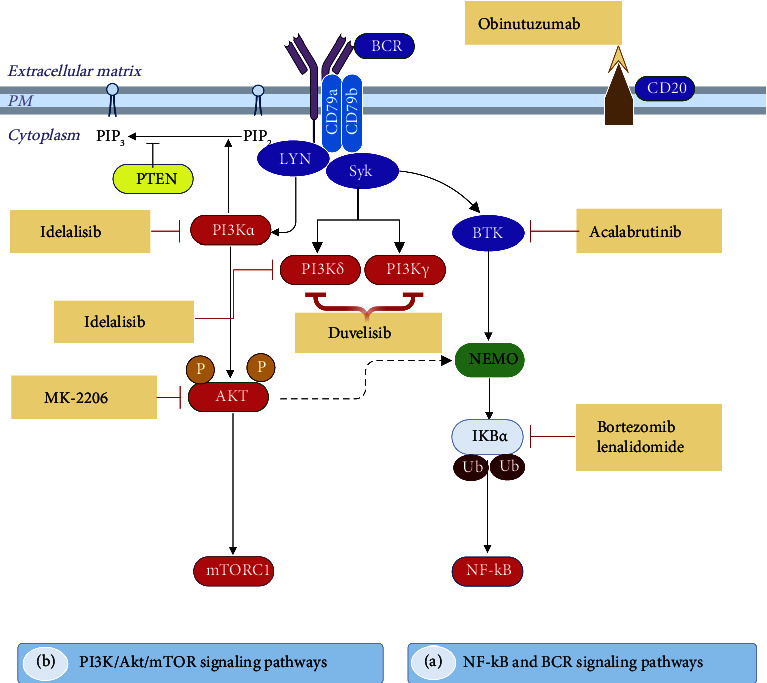
The therapeutic interventions that specifically target signaling pathways in B-cell lymphoma. The figure illustrates the interconnected network of (a) NF-*κ*B, BCR, and (b) PI3K signaling pathways, which provide promising therapeutic targets for the treatment of lymphoma. AKT, protein kinase B; Lyn, tyrosine–protein kinase Lyn; mTORC1, mammalian target of rapamycin complex 1; NEMO, IKKγ; NF-*κ*B, nuclear factor-kappa-B; PI3K, phosphoinositide 3-kinase; PIP2, phosphatidylinositol-(4,5)-bisphosphate; PIP3, phosphatidylinositol-(3,4,5)-trisphosphate; PTEN, phosphatase and tensin homolog; SYK, spleen tyrosine kinase; Ub, ubiquitination.

Determining the activation of oncogenic signaling pathways and biomarkers is critical for developing personalized, highly effective therapies for patients. Consequently, physicians, pharmacists, and researchers have recently been particularly interested in personalized medicine approaches and potential biomarkers for the selection of targeted medication. This section outlines the targeted medicines currently in clinical use and those in development, including ibrutinib, acalabrutinib, zanubrutinib, copanlisib, duvelisib, and idelalisib (Figure 2), which aim to disrupt the BCR signaling pathway, along with other inhibitors (refer to Table 2). We direct the reader to the most recent information on the mechanisms of action, effectiveness evidence, clinical trial phases, and potential adverse reactions of therapeutic medicines targeting critical signaling pathways implicated in B-NHL.

### 4.1. Targeting the BCR Signaling Pathway

BTK-driven pathways play a big role in the survival of cancerous B cells and the growth of tumors in different types of NHL, even when they are not activated normally [58]. A BTK inhibitor is a novel pharmacological agent demonstrating potential effectiveness in BCLs. Turning on BTK initiates downstream signaling pathways that drive cell division, growth, survival, motility, cytokine production, and antigen presentation. The direct downstream transfer of signals from BCR to BTK makes it an attractive target for inhibition through precise pharmacological design. The Food and Drug Administration (FDA) has authorized the use of two oral BTK inhibitors, ibrutinib, acalabrutinib, and zanubrutinib, to date now (Table 2) [59].

#### 4.1.1. Current Therapies

##### 4.1.1.1. Ibrutinib

Ibrutinib is classified as a tyrosine kinase inhibitor. BTK inhibitors represent a category of small-molecule drugs that impede B-cell proliferation and survival through irreversible binding to the protein BTK. Inhibiting BTK obstructs the BCR pathway, which frequently exhibits abnormalities. The US FDA swiftly approved ibrutinib for treating CLL/SLL, MCL, LPL/WM, and MZL in 2013, 2014, 2015, and 2017, respectively. Ibrutinib monotherapy has been shown to be sufficient to provide a satisfactory response rate (RR) in several subtypes of BCLs, including DLBCL [60] and FL [61].

Mechanism of Action: Ibrutinib is a potent, selective, and irreversible small-molecule inhibitor of BTK, administered orally. A cysteine residue (CYS481) forms a covalent bond with the active site of BTK. This inhibits the enzyme's activity. Ibrutinib inhibits the complete activation of BTK by obstructing its autophosphorylation at Tyr-223. This inhibition prevents downstream activation of the BCR pathway, thereby impeding the growth, proliferation, and survival of malignant B cells [62].

Clinical Data: Preclinical studies demonstrate significant efficacy and safety of ibrutinib in lymphoma models, including relapsed MCL [63], relapsed high-risk CLL [62], and other types of NHL: DLBCL [60], FL [61], MZL, and WM [64]. Common adverse events (AEs) reported included diarrhea, tiredness, dyspnea, and nausea [65]. Conversely, Mato et al. found that ibrutinib is commonly associated with off-target AEs such as atrial fibrillation, bleeding, arthralgia, diarrhea, and infection [66].

Studies by Caldeira et al. and Caron et al. have found a link between ibrutinib and AEs such as atrial fibrillation and hemorrhage. Compared to alternative therapies, patients with ibrutinib have a higher risk ratio for atrial fibrillation and high blood pressure, as well as a higher relative risk of overall bleeding. These findings highlight the potential risks associated with ibrutinib [67, 68]. In addition, a recent meta-analysis study encompassing 1436 studies confirmed that single-agent ibrutinib is associated with a significant risk of bleeding, diarrhea, and nausea. People with relapsed or refractory (R/R) MCL who received ibrutinib showed overall response rates (ORRs) ranging from 62.7 percent to 93.8 percent. In contrast, the ORRs for combinations involving ibrutinib ranged from 74% to 88%. Interestingly, in patients with newly diagnosed MCL receiving ibrutinib plus rituximab, ORRs varied between 84% and 100%. Patients treated with ibrutinib and rituximab exhibited the highest progression-free survival (PFS). Combining ibrutinib with other drugs may enhance its effectiveness and reduce the damage chemotherapy inflicts on cells, but it also necessitates close monitoring of side effects [69]. Consequently, certain AEs lead to the discontinuation of ibrutinib. Instead, combining ibrutinib with other drugs may improve its effectiveness and lessen the damage chemotherapy causes to cells, but it also requires careful monitoring of side effects; further research is necessary.

##### 4.1.1.2. Acalabrutinib

Acalabrutinib is classified as a tyrosine kinase inhibitor. Acalabrutinib represents a selective, next-generation inhibitor of BTK. This protein is part of the TEC family of nonreceptor protein kinases. Hematopoietic cells contain this protein, which aids in the functioning of the BCR signaling cascade. Acalabrutinib obtained FDA-accelerated approval in 2017 for the treatment of patients with MCL who had undergone one or more prior therapies. It was approved by the US FDA in November 2019 for the treatment of adult patients with CLL/SLL, based on two Phase III clinical trials [70]. Clinical trial phase studies are underway for WM, DLBCL, FL, and MZL [58].

Mechanism of Action: Acalabrutinib is a strong and selective covalent inhibitor of BTK. Compared to ibrutinib, it has less activity against other targets and a narrower range of kinase inhibition in kinome analysis. Acalabrutinib contains a reactive butynamide group that covalently binds to Cys481 in BTK. Acalabrutinib has less intrinsic reactivity than other BTK inhibitors. This means that it does not block as many off-target kinases that have the ability to bind cysteine-mediated covalently. Notably, off-target kinases, including epidermal growth factor receptor (EGFR) and interleukin 2–inducible T-cell kinase (ITK), were not subject to inhibition. Acalabrutinib is a strong BTK inhibitor, as shown by its ability to stop anti-immunoglobulin M from increasing CD69 expression in human peripheral blood mononuclear cells and whole blood [71].

Clinical Data: A Phase I/II preclinical and clinical studies demonstrate a correlation between the level and duration of BTK occupancy and in vivo efficacy. Healthy adult volunteers evaluated the pharmacokinetic properties of acalabrutinib, revealing rapid absorption and swift elimination [71]. Clinical trial, which is still going on, was set up to learn more about the safety, effectiveness, pharmacokinetics, and pharmacodynamics of acalabrutinib in people with CLL. This report's preliminary findings showed acalabrutinib's excellent BTK selectivity. Contrary to ibrutinib, acalabrutinib does not inhibit TEC kinase or platelet aggregation, lowering the risk of bleeding. Apart from that, acalabrutinib could reduce severe diarrhea and skin rashes, even though it does not block EGFR. However, more patients and a longer follow-up period are needed to determine the benefits and drawbacks of this research [72]. A Phase II study of acalabrutinib for patients with R/R MCL involved 124 participants who were administered acalabrutinib at a dosage of 100 mg twice daily (BID). The ORR was 81 percent, with a CR rate of 40%, indicating that the study achieved its primary endpoint. Headache (38 percent), diarrhea (36 percent), tiredness (28 percent), cough (22 percent), and myalgia (21 percent) were the most frequent side effects. The majority of these AEs were classified as Grades 1–2, manifested early in the treatment process, and were manageable [73].

In six clinical studies with a total of 610 patients, 98.9% of participants had AEs, with 73% reporting treatment-related ones. Headaches, diarrhea, tiredness, nausea, and bruises were Grade ≥ 3 AEs. 35.7 percent of participants reported safety incidents (SAEs), and 9.5 percent related to the study therapy. Acute atrial fibrillation occurred in 2.3% of patients, 12 of whom had risk factors. 61.0% of dyspnea had infections. Infrequent AEs caused discontinuations (6.1%), with pneumonia, thrombocytopenia, anemia, dyspnea, and neutropenia being the most common. In MCL patients, AEs caused 1.6% dosage reductions and 6.5% discontinuations. In the ELEVATE-TN study, 11% of patients discontinued therapy due to AEs and 7% decreased dosage. AEs caused 11% of ASCEND study subjects to discontinue, mostly secondary primary cancers or infections. AEs caused 34% and 4% of dose interruptions and reductions, respectively. MCL and CLL patients had a few AEs that necessitated dosage reductions or discontinuations across studies [58]. Acalabrutinib has been shown to be very effective and safe for people with MCL and CLL. It has also shown promise in helping people who are unable to tolerate ibrutinib. The toxicity profiles of acalabrutinib and ibrutinib are not feasible due to the lack of a direct comparison, but acalabrutinib showed lower side effects. On the other hand, the combination of acalabrutinib and venetoclax (VTC) shows significant promise, with preliminary data from ongoing trials, indicating that this treatment is well-tolerated. Subsequent data from these studies will ascertain its integration into treatment guidelines.

##### 4.1.1.3. Zanubrutinib

Zanubrutinib is a novel, oral BTK inhibitor similar to other second-generation BTK inhibitors. It is a highly selective, irreversible BTK inhibitor that has demonstrated significant results in lymphoid malignancies in early-phase research. The development of zanubrutinib aimed to improve BTK selectivity, potentially providing tolerability advantages over ibrutinib, particularly in terms of treatment-limiting toxicities caused by the inhibition of off-target tyrosine kinases. In November 2019, zanubrutinib received approval. It is a strong and long-lasting BTK inhibitor that has shown profound and long-lasting effects in WM, CLL, and MCL. In addition, the FDA approved zanubrutinib through the accelerated approval pathway to treat patients with MZL who have already received one anti-CD20-based therapy and patients with MCL who have already received one or more therapies [74]. People with R/R MZL and FL are currently in the clinical trial phase for zanubrutinib as a potential useful addition to current treatments [75].

Mechanism of Action: Zanubrutinib inhibits tumor progression and stops neoplasm B-cell proliferation. Preclinical and clinical studies have demonstrated zanubrutinib's ability to combat cancer in CLL. It does this by blocking ITK and EGFR in vitro. T-cell exhaustion is restored by blocking checkpoint molecules on suppressor cells and adhesion receptors on B cells. This controls the immune microenvironment. Higher doses of zanubrutinib diminish the role of NK cells in enhancing rituximab-dependent cytotoxicity. Zanubrutinib was better at targeting BTK and had fewer effects on other cells and enzymes than ibrutinib in a number of different tests [76].

Clinical Data: Xu et al. evaluated the long-term follow-up results of zanubrutinib in multiple lines of treatment in patients with CLL/SLL. With longer follow-up, zanubrutinib provided long-term benefits and a favorable safety profile for individuals with TN or RR CLL/SLL. Early use of zanubrutinib was associated with improved results (Xu et al., 2022). Zanubrutinib represents an effective and well-tolerated therapeutic option for R/R MCL. Early intervention with zanubrutinib is associated with improved survival outcomes [77]. The reported safety and effectiveness results [75, 77–79] show a good benefit-risk profile, which supports the idea that zanubrutinib could be a clinically important addition to current NHL treatments.

#### 4.1.2. Emerging Therapies

##### 4.1.2.1. Tirabrutinib

Tirabrutinib irreversibly binds to BTK in B cells. This stops abnormal BCR signaling that happens in B-cell cancers and autoimmune diseases. Clinical development is advancing in the United States, Europe, and Japan for autoimmune disease, CLL, B-lymphoma, Sjögren's syndrome, pemphigus, and rheumatoid arthritis. In March 2020, Japan approved oral tirabrutinib to treat recurrent or refractory primary central nervous system lymphoma. In Japan, tirabrutinib is currently under regulatory review to treat WM [80].

##### 4.1.2.2. Orelabrutinib

Orelabrutinib is a novel small molecule that acts as a selective irreversible inhibitor of BTK. Orelabrutinib demonstrates significant efficacy and an acceptable safety profile in patients with R/R Waldenström's macroglobulinemia (R/R WM) [81]. The Japan Pharmaceuticals and Medical Devices Agency and the China Food and Drug Administration, respectively, have approved orelabrutinib for use in 2020 [82].

#### 4.1.3. Combination Therapies

##### 4.1.3.1. Loncastuximab Plus Ibrutinib

The combination of loncastuximab, tesirine, and ibrutinib has shown promising results. The preliminary findings of the Phase I/II study are promising. The first results shown at the ASH 2020 conference showed that in people with R/R MCL, the ORR was 100% (3 out of 3 patients), and the CR percentage was 33.3% (1 patient). The updated results indicated an ORR of 85.7% in the cohort of seven patients with R/R MCL [83]. Furthermore, there are promising combinations; see Table 3 for references.

### 4.2. Targeting the PI3K–AKT–mTOR Pathway Inhibitors

The PI3K pathway is crucial to cell survival, proliferation, and differentiation. Thus, PI3K is a major therapeutic target for various human malignancies, including B-NHLs. Four isoforms of P13K catalytic domains exist: p110*α*, p110β, p110γ, and p110*δ*. Many tissues use different signaling pathways for each isoform. Among all isoforms, the p110*δ* isoform is dominant in lymphoid cells and is important for B-NHL signaling. The US FDA approved idelalisib, duvelisib, and copanlisib as PI3K inhibitors. Clinically, PI3K isoform overexpression predicts poor survival. The overactive PI3K signaling pathway in B-cell neoplasms makes chemotherapy less effective, which makes it a good target for treatment.

#### 4.2.1. Current Therapies

##### 4.2.1.1. Idelalisib

Idelalisib is a purine quinazoline derivative that can be taken by mouth. It was clinically developed as a first-in-class targeted treatment for CLL and associated B-cell lymphoproliferative diseases [100]. Idelalisib is a first-in-class PI3K inhibitor that obtained accelerated approval from the US FDA in July 2014 for use as a monotherapy in relapsed FL and SLL [100].

Mechanism of Action: Idelalisib specifically targets P110*δ*, the delta isoform of the enzyme PIP2 3-kinase, commonly referred to as PI3K*δ*. Idelalisib promotes apoptosis and suppresses proliferation in cell lines originating from malignant B cells, as well as primary tumor cells. Additionally, it blocks several cell signaling pathways, such as the BCR signaling pathway and the CXCR4 and CXCR5 pathways. These pathways help B cells move and find their way to the lymph nodes and bone marrow. The use of idelalisib on lymphoma cells resulted in reduced chemotaxis and adhesion, as well as a decrease in cell viability [101].

Clinical Data: Idelalisib, a phosphatidylinositol 3-kinase *δ* inhibitor, is authorized to treat R/R FL, indolent NHL, and CLL. Treatment interruption and dosage decreases can help control AEs, perhaps prolonging therapy duration and enhancing RRs. Post hoc analyses revealed that patients with indolent NHL who encountered ≥ 2 interruptions had longer PFS and overall survival than CLL patients with ≥ 2 interruptions. Patients with CLL who stopped using idelalisib for > 6 months still had clinical improvements. Interrupting treatment for more than 8% of the time may reduce its therapeutic benefits [93]. Unexpectedly, serious side effects including Grade 4 sepsis, hypotension, and lung infection were described in 2 Alliance Phase I trials of idelalisib, lenalidomide, and rituximab for R/R FL and MCL. More serious AEs linked to idelalisib have been identified by longer follow-up data, resulting in a black box warning on prescription instructions [102].

In 2016, we discontinued three RCTs due to increased mortality and severe deleterious side effects in CLL or indolent NHL: idelalisib plus rituximab versus placebo plus rituximab in untreated CLL; idelalisib plus rituximab versus placebo plus rituximab in R/R indolent NHL; and pooled analysis of idelalisib groups versus control: fatalities (7.4% vs. 3.5%) and overall survival HR 2·29 (95% CI 1.26–4.18). In 2022, Gilead Sciences, Inc., voluntarily withdrew the idelalisib indications for FL and SLL, citing inadequate enrollment in confirmatory studies. Subsequent withdrawals of next-in-class PI3K agents occurred due to increased safety concerns regarding the entire class of drugs [103]. For more information, we refer the reader to other references [104].

##### 4.2.1.2. Duvelisib

Duvelisib (IPI-145) is an oral PI3K*δ*/γ inhibitor. Inhibiting both PI3K*δ* and γ may enhance anticancer effectiveness, since PI3Kγ is expressed in supportive cells such CD4+T cells and tumor-associated macrophages [105]. In 2018, the FDA approved duvelisib for R/R CLL/SLL and FL [106].

Mechanism of Action: Duvelisib is a strong, reversible PI3K inhibitor that targets the gamma and delta forms of the enzyme (PI3K*δ*/γ). PI3K is critical in both innate and adaptive immunity, and inhibiting the delta and gamma forms is important for immune suppression. Hematopoietic cells confine the activities of PI3K gamma and delta, which are essential for normal B-cell development. Enhanced PI3K activation in lymphomas enables unrestricted growth and survival. Therefore, inhibition of PI3K can impede BCR signaling, disrupt cytokine signaling from the microenvironment, and enhance antitumor immunity.

Clinical Data: The Phase I trial looked at the safety and effectiveness of duvelisib as a single treatment for iNHL that was not responding to rituximab, chemotherapy, or radioimmunotherapy. The open-label, global Phase II study involved 129 (SLL, FL, and MZL) patients and yielded an ORR of 47.3%. Duvelisib may be a novel oral therapeutic option for the elderly who require additional medications [92]. In both duvelisib and idelalisib, immune-related AEs were the most frequent cause of discontinuation, interruption, or dose reduction of duvelisib, whereas diarrhea was the most common AE in duvelisib-treated patients [73]. As with previous studies, oral duvelisib monotherapy in heavily pretreated, double-refractory NHL showed activity that was clinically relevant and safety that was tolerable [92]. Duvelisib effectively treats R/R mature lymphocyte neoplasms. Duvelisib has greater efficacy in treating iNHL than FL and other approved PI3K inhibitors. Duvelisib has the potential to reduce the risk of fatal and severe toxicity. For more information, we refer the reader to other references [107].

##### 4.2.1.3. Copanlisib

Copanlisib (BAY 80-6946) is a small-molecule inhibitor of PI3K that works specifically on four key isoforms. It is especially effective against PI3K*α* and PI3K*ε*, which are important in B-cell cancers [108]. In September 2017, the FDA approved copanlisib for the treatment of adult patients with relapsed FL [109].

Mechanism of Action: The PI3K pathway is recognized as one of the most frequently activated signaling pathways in various cancers and has demonstrated the ability to rescue mature B cells that are deficient in BCRs, resulting in their proliferation. In B-cell malignancies, including FL, overexpression of PI3K isoforms predicts poor prognosis and contributes to relapse and cancer resistance [110]. Copanlisib stops four types of class 1 enzymes from working. These are PI3K*α*, PI3Kβ, PI3Kγ, and PI3K*δ*. PI3K*α* and PI3Kβ exhibit widespread expression across various cell types, whereas PI3Kγ and PI3K*δ* are predominantly expressed in hematopoietic tissues. PI3K*δ* is associated with B-cell proliferation and survival, whereas PI3K*α* is linked to relapsed disease. This offers a significant opportunity for tumor management through PI3K inhibition. Unintentional beneficial effects include enhancing apoptotic pathways, halting CXCR12-mediated chemotaxis in malignant B cells, and inhibiting NF-B signaling in lymphoma cell lines [110].

Clinical Data: Lenz et al. investigated the efficacy and safety of copanlisib, a PI3K inhibitor, in patients with R/R DLBL. The main endpoints were the objective response rate in DLBCL COO subgroups and the CD79B mutation status. The study discovered that the ORR was 19.4% in ABC- and GCB-DLBCL patients and 22.2%–20.0% in patients with and without CD79B mutations. Hypertension, diarrhea, and hyperglycemia were some of the AEs. Researchers found aberrations in 338 genes and discovered a 16-gene signature that distinguishes responders from nonresponders. Copanlisib therapy has a reasonable safety profile for individuals with R/R DLBCL [88]. Copanlisib seems to be a safer option than idelalisib and duvelisib, which have substantial side effects. As a result, the efficacy and safety of copanlisib must be compared and studied alongside those of other lymphoma treatments. For more information, we refer the reader to other references [111].

#### 4.2.2. Emerging Therapies

##### 4.2.2.1. MK2206

The first allosteric AKT inhibitor in clinical development, MK-2206, inhibits AKT1 and AKT2 at nanomolar levels. MK-2206 stopped AKT and cell growth in several types of human cancer cells, and it may be able to beat mTOR inhibitor resistance in BCL. Oki et al. tested an AKT-targeted lymphoma drug initially. The AKT inhibitor MK2206 induced responses in cHL and indolent lymphoma, indicating additional research. Against DLBCL, T-cell lymphoma, and MCL, single-agent activity is modest. MK2206 treatment may have raised cytokine levels, which may explain this lack of response. Alternative survival pathways or inadequate AKT phosphorylation at low dosages may also cause AKT inhibitor resistance [87].

##### 4.2.2.2. CUDC-907

CUDC-907, an oral small-molecule inhibitor of HDAC and PI3K enzymes, has shown antitumor activity in preclinical models, including those driven by MYC [112]. The clinical trial study by Oki et al. showed that CUDC-907, with or without rituximab, demonstrated similar safety profiles in patients with R/R DLBCL, with a particular focus on those with MYC-altered disease. The study involved 37 DLBCL patients, with 14 having confirmed MYC-altered disease. The most frequently reported Grade ≥ 3 treatment-related events were thrombocytopenia, neutropenia, diarrhea, fatigue, and anemia. The RR was 37% in evaluable patients and 64% in MYC-altered patients. The median duration of response was 11.2 months. Because CUDC-907 is safe and has been shown to have long-lasting antitumor effects, it should continue to be developed for these groups with high unmet needs [90].

#### 4.2.3. Combination Therapies

##### 4.2.3.1. Copanlisib With Rituximab

Copanlisib is the first PI3K inhibitor that has been shown to be safe, well-tolerated, and effective when used with immunochemotherapy. It is still being tested on people who have relapsed indolent BCL. The Phase III safety run-in included patients with relapsed indolent lymphoma who had experienced relapse after 1 to 3 lines of treatment. Copanlisib was administered to 10 patients receiving rituximab and bendamustine (R-B) and to 11 persons receiving R-CHOP. Both groups saw similar AEs in the copanlisib monotherapy studies. Complications included a decreased neutrophil count, nausea, reduced platelet count, elevated blood sugar, and hypertension. Preliminary findings indicated that copanlisib combined with R-B and R-CHOP achieved objective response rates of 90% and 100%, respectively. Copanlisib, the first PI3K inhibitor, is safe, acceptable, and efficacious when combined with immunochemotherapy for relapsed indolent BCL [113]. In summary, making effective inhibitors that target mutant PI3K isoforms and using strategic combination methods could help lower side effects and improve effectiveness.

### 4.3. BCL2 Inhibitors in Apoptosis Pathway

Apoptosis is initiated more rapidly in the intrinsic apoptotic pathways because of hypoxia, DNA damage, and oncogenic stress. The control and transduction of the signals in this type of pathway are done by the interaction of BCL2 family members, DNA checkpoint proteins, and p53, which are situated in the downstream as well as upstream cascades. This finally helps in the activation of caspase 3 and caspase 7 for producing extrinsic pathway-like effects [25]. Recently, “high-grade BCL with MYC and BCL2 and/or BCL6 rearrangements” (HGBL-DH) was officially classified as a distinct entity by the WHO. Numerous cancer centers recommend dose-intensive chemotherapy regimens due to the extremely poor prognosis associated with these lymphomas [50]. The development of an inhibitor of BCL2 is considered an optimal target for therapeutic development of malignant lymphomas.

#### 4.3.1. Current Therapies

##### 4.3.1.1. VTC

VTC is an oral B-cell lymphoma-2 (BCL-2) inhibitor indicated. Inhibitor VTC is an orally administered medication known for its relatively safe administration route [38]. On April 11, 2016, the US FDA approved VTC (VENCLEXTA tablets, sold by AbbVie, Inc., and Genentech USA, Inc.) as a treatment for CLL in people who have had at least one previous treatment and have been diagnosed with 17p deletion using an FDA-approved test [114].

Mechanism of Action: VTC is a selective BCL-2 inhibitor that works by interacting directly with the BCL-2 protein. This changes the mitochondrial apoptosis pathway and helps tumor cells die. It has proven effective in a variety of hematological malignancies. VTC is more selective for BCL-2. This lowers the risk of thrombocytopenia and leads to better therapeutic outcomes with fewer severe side effects [115].

Clinical Data: VTC shows promise for people with lymphatic system cancers that have not responded to other treatments [116, 117]. Ma et al. reported poor outcomes in individuals with DLBCL who overexpressed BCL-2 protein. The clinical trial study employed population pharmacokinetics and exposure–response analysis to confirm the dosage chosen for further research. A total of 216 patients with R/R or 1L NHL participated in the study, receiving eight 21-day cycles of 400–800 mg VTC, along with R for eight cycles and CHOP for 6–8 cycles. There was no significant relationship between VTC AUCs and PFS or CR. The study also found that the dose intensities for VTC and R-CHOP components were the same across all VTC exposures. This suggests that VTC did not change the delivery of R-CHOP backbone [91]. VTC, a fixed-duration combination therapy with anti-CD20 monoclonal antibodies, promotes significant and frequent remissions in CLL/SLL and is currently the standard of care for relapsed and frontline diseases. Apart from CLL/SLL, VTC has demonstrated significant potential in treating MCL and WM, a condition where successful targeted therapy combination regimens are likely to incorporate it. More research needs to be done on therapies that target other members of the BCL2 family, biomarkers that have been clinically proven to be sensitive to BH3-mimetic drugs, and new ways to combine drugs to make them more effective. For more information, we refer the reader to other references [118].

#### 4.3.2. Emerging Therapies

##### 4.3.2.1. BM-1197

BM-1197 is a small-molecule compound that has a structure similar to BH3 and binds very strongly to Bcl-2 family proteins. Through an endogenous apoptotic pathway, BM-1197 induces apoptosis. Sun et al. showed that BM-1197 has strong antitumor effects both in vitro and in vivo. This provides encouraging preclinical data for further development of BM-1197 in malignant lymphoma [119]. Their findings suggest that BM-1197 could be a potential therapeutic agent for the treatment of malignant lymphoma. To fully understand the mechanisms of action and potential side effects of BM-1197 in clinical settings, further research is necessary.

### 4.4. The NF-*κ*B Signaling Pathway Target Remedies

For the past 30 years, academia and the pharmaceutical industry have concentrated on targeting the key components of the NF-*κ*B signaling pathway. This extensive research initiative has resulted in the creation of multiple therapeutic strategies capable of modulating the NF-*κ*B core pathway [120].

#### 4.4.1. Emerging Therapies

##### 4.4.1.1. Bortezomib

The ABC subtype of DLBCL is more likely to have mutations in genes related to BCR signaling and NF-*κ*B regulation. Bortezomib, a proteasome inhibitor, can inhibit NF-*κ*B activity by blocking the degradation of the inhibitor I*κ*B*α*. Bortezomib, when paired with infusional chemotherapy, has demonstrated selective efficacy in DLBCL subtypes. The PFS rate for patients with double-hit lymphoma appears to be higher than in previous trials, and this is consistent with more recent analysis. Almost half of double-hit lymphomas did not develop after 30 months. At 30 months, the PFS rate in patients with double-hit lymphoma was 38.9% after R-CHOP versus 58.8% after RB-CHOP [85]. Dunleavy et al. conducted a clinical trial to assess the safety and efficacy of bortezomib (PS-341), an experimental drug, when administered alone or in combination with a chemotherapy regimen. The study showed strong evidence that combining bortezomib with chemotherapy is an effective way to treat ABC-DLBCL, which is the subtype that is hardest to cure with the standard treatment, R-CHOP. These results provide the important clinical evidence needed to support a randomized study of R-CHOP with and without bortezomib in people with ABC-DLBCL who have not been treated yet. Moreover, they express concern over the effectiveness of bortezomib in GCB-DLBCL and advise exercising caution while using this medication in this specific subtype.

##### 4.4.1.2. Lenalidomide

Lenalidomide is an oral immunomodulatory agent. It works on NHL cells in both direct and indirect ways, and it works well as a single agent in aggressive and indolent B-cell NHL that has come back or stopped working, such as MCL, DLBCL, and FL [121]. Lenalidomide modulates the immune system by imparting pleiotropic antitumor effects such as antiangiogenesis and immune modulation. It also restricts the hyperactivity of NF-*κ*B signals in carcinomas and shows efficiency in individuals suffering from MCL and FL [122]. In DLBCL, combining ibrutinib and lenalidomide increases activity, especially in non-GCB and ABC-DLBCL, which depend on BCR and MYD88 signaling pathways, NF-*κ*B activation, and upregulation of interferon regulatory factor 4 expression for survival. BCLs may resist rituximab; however, lenalidomide's immunomodulatory properties may help. Ibrutinib and rituximab may help MCL [34].

### 4.5. Other Pathways and Novel Approaches

Blocking the Notch pathway can either directly or indirectly affect the cancerous cells. It suppresses the tumor growth by decreasing the supply of blood to the cancerous cells as a result of angiogenesis dysregulation and by disrupting tumor-forming stem cells. This became the basis for the therapeutic approaches used in cancer-inhibiting strategies. Also, it is a well-known fact that tumor cells sometimes go through many mutational processes in various cascades interconnected with Notch (WNT and Hedgehog), which suggest the usage of target-based remedies when administered in combined form. Combining Notch inhibitors with traditional chemoradiotherapy is also found to be effective because of the enhanced curative effect when administered in combined form. Many Notch-targeting strategies, such as inhibitors of *α*-secretase (ASIs) and γ-secretase (GSIs), immunotherapeutic agents along with neutralizing antibodies that target either ligands or Notch receptors, and using soluble recombinant receptors and ligands as traps are currently under development [123].

#### 4.5.1. CB-103 (CSL-NICD Inhibitor)

A new orally active small-molecule inhibitor (CB-103) targeting the NOTCH pathway was discovered and developed in clinical trial. CB-103 inhibits NOTCH signaling in a special way that targets the NOTCH transcriptional activation complex. For more information, we refer the reader to other references [124].

#### 4.5.2. Tazemetostat (EZH2 Inhibitor)

Tazemetostat is a pharmacological agent that inhibits the EZH2 protein to prevent lymphoma cells from proliferating. People who had already received extensive treatment for FL but had relapsed or refused to respond to other treatments found it effective as a single [42]. The open-label, single-arm Phase II study was conducted in 38 clinics or hospitals in France, the UK, Australia, Canada, Poland, Italy, Ukraine, Germany, and the United States. Adults with histologically confirmed FL who had relapsed or were resistant to two or more systemic treatments and had enough tumor tissue for central EZH2 mutation testing were considered eligible. Patients received 800 mg of tazemetostat orally, administered twice daily for a duration of 28 days. The objective response rate, determined according to the 2007 International Working Group criteria for NHL, was the main outcome. Tazemetostat monotherapy produced clinically significant, long-lasting responses and was usually well tolerated in strongly pretreated individuals with R/R FL [42]. Tazemetostat is a novel FL treatment with clinically relevant and sustained responses, excellent safety, and a unique mode of action. The US FDA has granted approval for tazemetostat for patients with FL who have undergone a minimum of two prior treatments and possess EZH2 mutations, or for those with FL lacking alternative therapeutic options. The drug remains unapproved in Europe.

## 5. Conclusion and Future Direction

Lymphoma patients, particularly those with R/R BCL, have a promising outlook because of the use of pathway-based targeted treatment in combination with immunotherapy or conventional chemotherapy. Therefore, in the realm of personalized medicine, identifying the activation of cancer-causing pathways and signatures is crucial and beneficial, enabling the integration of personalized medicine targeting these pathways into patients' treatment regimens.

The approval and development of novel targeted therapies have marked recent advancements in the B-NHL therapy landscape. Individuals with R/R DLBCL have experienced a limited number of effective targeted therapies. The United States, Europe, and various other nations have authorized chimeric antigen receptor (CAR-T) T-cell therapy [125]. However, there are various obstructions that impede individuals from receiving this treatment, such as the difficult management of significant CAR-T-related toxicities, a restricted availability of appointments, and a limited number of treatment facilities. An alternative approach involves a chemotherapy-free combination treatment utilizing a BTK inhibitor alongside other medications for a specified duration. Ibrutinib and VTC demonstrated efficacy in untreated B-CLL patients over a defined 24-cycle duration [126]. Combining treatments may deepen remissions, reduce resistance, and eliminate aggressive clones. An effective strategy allows for a specified therapy duration and might decrease the risks and expense of long-term targeted drug exposure.

Recent clinical research has led to the development of personalized medications for B-NHL, providing patients with multiple advantageous therapeutic options. Nonetheless, specific toxicities require attention. Identifying the source of toxicity and developing the most effective treatment strategy are essential. Despite employing advanced therapeutic techniques, treatment resistance remains a significant challenge. Consequently, a thorough understanding of the in vivo mechanisms of action of these drugs is essential; further research is necessary.

## Figures and Tables

**Table 1:** Summary of genetic alterations in B-NHL-related dysregulated signaling.

B-cell lymphoma	Genetic alterations	Refs.
Diffuse large B-cell lymphoma (ABC-DLBCL)	BCL6, BCL10, CARD11, TP53, MYC, KMT2D, CREBBP, EP300, CD58, BCL2, TNFAIP3, MYD88, CD79B, PRDM1, NOTCH1, MYC	[29–35]
Diffuse large B-cell lymphoma (GCB-DLBCL)	BCL6, BCL10, CARD11, TP53, MYC, KMT2D, CREBBP, EP300, CD58, BCL2, TNFRSF14, MIR17HG, REL, S1PR2, GNA13, EZH2	

Chronic lymphocytic leukemia (CLL)	*NOTCH1, SF3B1, ATM, BIRC3, NFKBIE, EGR2, MYD88, XPO1, CHD2,* BTK, PLCG2, BCL2, TP53, CARD11	[33, 36–39]

Burkitt lymphoma (BL)	MYC, TP53, TCF3, ID3, CDKN2A, DDX3X	[32, 40]

Marginal zone lymphoma (MZL)	KLF2, NOTCH2, TP53, TNFAIP3, KMT2D, KMT2D, CREBBP, Fas, KLF2, NOTCH1, NF1, TRAF3, and ATM	[33, 41]

Follicular lymphoma (FL)	KMT2D, CREBBP, EP300, and EZH2; TNFRSF14, CARD11, TNFAIP3, CD79A/B; and MYD88, BCL2, TP53, RRAGC, NOTCH2, DTX1, SOCS1, STAT6, and STAT3	[42–44]

Mantle cell lymphoma (MCL)	TP53, ATM, KMT2A, MAP3K14, BTK, TRAF2, CHD2, TLR2, ARID2, RIMS2, NOTCH2, TET2, SPEN, NSD2, CARD11, CCND1, SP140, CDKN2A, and S1PR1	[33, 45–47]

**Table 2:** Approved and developmental BTK inhibitors in B-NHLs.

	Current therapies			Emerging therapies		
	Ibrutinib	Acalabrutinib	Zanubrutinib	Spebrutinib	Tirabrutinib	Orelabrutinib
Approved (US FDA)	CLL, MCL, WM, MZL	MCL, CLL	MCL	—	PCNSL, 2020†	WM, 2020†
Development (clinical trials)	DLBCL, FL	WM, DLBCL, FL, MZL	WM, CLL, FL, MZL	B-NHL, CLL, WM	B-NHL, CLL, WM	—

Abbreviations: B-NHL, B-cell non-Hodgkin lymphoma; CLL, chronic lymphocytic leukemia; DLBCL, diffuse large B-cell lymphoma; FL, follicular lymphoma; MCL, mantle cell lymphoma; MZL, marginal zone lymphoma; PCNSL, primary central nervous system lymphoma; WM, Waldenstrom macroglobulinemia.

^†^Approved by Japanese and China FDA.

**Table 3:** Targeted therapies developed for the signaling pathway in B-NHLs.

B-NHL	Targeted signaling	Intervention drug	Recruitment status	Clinical trial no.	Ref.
Diffuse large B-cell lymphoma, (DLBCL)	NF-*κ*B	Lenalidomide	Completed	NCT01122472	[84]
		Bortezomib	Completed	NCT01324596	[85]
	BTK	Ibrutinib with lenalidomide + rituximab	Completed	NCT02077166	[34]
		Ibrutinib, obinutuzumab, and venetoclax	Active	NCT02558816	[86]
	AKT	MK-2206	Completed	NCT01258998	[87]
	PI3K	Copanlisib	Completed	NCT02391116	[88]
		Copanlisib + rituximab-bendamustine	Recruiting	NCT04433182	[89]
		CUDC-907	Completed	NCT01742988	[90]
	mTOR	Everolimus and lenalidomide	Completed	NCT01075321	
	BCL2	Venetoclax		NCT02055820	[91]

Chronic lymphocytic leukemia (CLL)	PI3K	Duvelisib	Completed	NCT01882803	[92]
		Idelalisib	Completed	NCT01282424	[93]
	BTK	Ibrutinib	Completed	NCT01744691	[94]
		Zanubrutinib		NCT03206918	[95]
		Acalabrutinib	Completed	NCT02029443	[72]
	NF-*κ*B	Lenalidomide	Completed	NCT00535873	[96]
	BCL2	Venetoclax + ibrutinib		NCT02910583	[97]

Marginal zone lymphoma (MZL)	BTK	Parsaclisib	Completed	NCT03314922	No result posted
	PI3K	Umbralisib	Terminated	NCT02793583	[98]

Follicular lymphoma (FL)	BCL2	Venetoclax + bendamustine + rituximab	Completed	NCT02187861	[44]
	EZH2	Tazemetostat	Completed	NCT01897571	[42]

Mantle cell lymphoma (MCL)	Akt	Lenalidomide + rituximab	Completed	NCT01472562	[99]
	BTK	Ibrutinib	Completed	NCT01236391	[65]
	Proteasome	Bortezomib + ruxolitinib	Completed	NCT02613598	

## Data Availability

The authors have nothing to report.
